# Enzyme Inhibition by Molluscicidal Components of *Myristica fragrans* Houtt. in the Nervous Tissue of Snail *Lymnaea acuminata*

**DOI:** 10.4061/2010/478746

**Published:** 2009-12-06

**Authors:** Preetee Jaiswal, Pradeep Kumar, V. K. Singh, D. K. Singh

**Affiliations:** Department of Zoology, D.D.U. Gorakhpur University, Gorakhpur 273009, India

## Abstract

This study was designed to investigate the effects of molluscicidal components of *Myristica fragrans* Houtt. (Myristicaceae) on certain enzymes in the nervous tissue of freshwater snail *Lymnaea acuminata* Lamarck (Lymnaeidae). *In vivo* and *in vitro* treatments of trimyristin and myristicin (active molluscicidal components of *Myristica fragrans* Houtt.) significantly inhibited the acetylcholinesterase (AChE), acid and alkaline phosphatase (ACP/ALP) activities in the nervous tissue of *Lymnaea acuminata*. The inhibition kinetics of these enzymes indicates that both the trimyristin and myristicin caused competitive noncompetitive inhibition of AChE. Trimyristin caused uncompetitive and competitive/noncompetitive inhibitions of ACP and ALP, respectively whereas the myristicin caused competitive and uncompetitive inhibition of ACP and ALP, respectively. Thus results from the present study suggest that inhibition of AChE, ACP, and ALP by trimyristin and myristicin in the snail *Lymnaea acuminata* may be the cause of the molluscicidal activity of *Myristica fragrans*.

## 1. Introduction

The snail *Lymnaea acuminata* Lamarck (Lymnaeidae) is the vector of liver flukes,* Fasciola gigantica *Cobbold (Fascioliodae) and* Fasciola hepatica* Linnaeus (Fascioliodae) which are responsible for endemic fascioliasis in cattle population of northern India [1, 2]. One way to reduce the risk of fascioliasis is to delink the life cycle of the flukes by killing the vector snail [3, 4]. Recently, it has been reported that* Myristica fragrans* (Myristicaceae) seed (nutmeg) and aril (mace) have potent molluscicidal activity against *Lymnaea acuminata* [5]. The active moieties responsible for the molluscicidal activity are trimyristin and myristicin [5]. The mechanism by which these active components cause snail death is not known. The present study is an extension of our previous study aimed at elucidating the effect of the active moieties on the different enzymes namely, acetylcholinesterase (AChE), acid phosphatase (ACP), and alkaline phosphatase (ALP) in the nervous tissue of snail *Lymnaea acuminata*. 

## 2. Materials and Methods

### 2.1. Test Material

Trimyristin (1,2,3-tritetradecanoylglycerol) catalog no. T 5141, and myristicin (4-methoxy-6-(2-propenyl)-1,3-benzodioxole), catalog no. M 9411, were purchased from Sigma Chemical Co., USA.


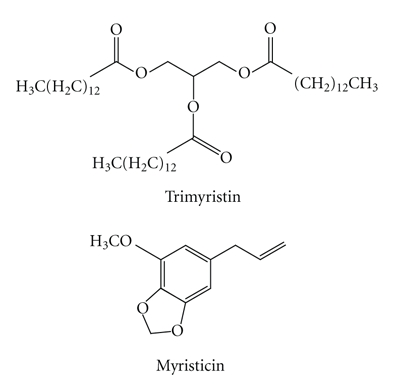


### 2.2. Bioassay

Adult *Lymnaea acuminata* (length, 2.25 ± 0.20 cm) were collected locally from Ramgarh Lake located almost adjacent to this University campus. Snails were acclimatized to laboratory conditions for 72 hours and used as experimental animals. Twenty experimental snails were kept in a glass aquarium containing 3 L of dechlorinated tap water at 22–24°C. Six aquaria were set up for each concentration. Each set of experimental snails was exposed to sublethal concentrations −40 and 80% of 24 h and 96 h LC_50_ of trimyristin (24 h- 6.21, 12.43 mg L^−1^; 96 hours- 2.80, 5.60 mg L^−1^) and myristicin (24 hours- 0.60, 1.20 mg L^−1^; 96 hours 0.06, 0.12 mg L^−1^) for 24 and 96 hours. These concentrations were based on 24 and 96 hours LC_50 _values earlier reported by us [5]. Control animals were kept in an equal volume of dechlorinated water under similar conditions without any treatment. After 24 and 96 hours of treatment snails were removed from the aquaria and rinsed with water. Nervous tissue, present around the buccal mass of the snail, was taken out for the measurement of AChE, ACP, and ALP activities in the treated and control groups of snails. 

*In vitro* experiments were performed by dissolving the molluscicides in ether and an appropriate volume containing 3, 5, 7, and 9 *μ*g of trimyristin and 0.3, 0.5, 0.7, and 1.0 *μ*g of myristicin (**0.04, 0.07, 0.09, 0.1, and 0.15** **mM **of trimyristin** and 0.01, 0.02, 0.03, 0.05, and 0.09** **mM** of myristicin) was added to 10 mm path length cuvette separately. Ether was then allowed to evaporate. Molluscicides were preincubated for 15 minutes at 25°C with an enzyme source and then enzyme activity was determined. The control cuvette contained ether only. The Michaelis-Menten constant (*K*_*m*_) and maximum velocity (*V*_max_) of different enzyme inhibitions were calculated by nonlinear regression. Lineweaver-Burk plots for the hydrolysis of different concentrations of substrate by the treated (0.09 mM trimyristin and 0.03 mM myristicin) and untreated enzymes were plotted to observe mode of enzyme inhibition [6]. IC_50_ values of myristicin and trimyristin were calculated in between the negative log concentration of inhibitor versus relative activity between inhibited and uninhibited enzymes.

### 2.3. Enzyme Assay

#### 2.3.1. Acetylcholinesterase

Acetylcholinesterase (AChE) activity was measured by the method of Ellman et al. [7] as modified by Singh and Agarwal [8]. 50 mg of nervous tissue of *Lymnaea acuminata* taken around the buccal mass was homogenized in 1.0 mL of 0.1 M phosphate buffer pH 8.0 for 5 minutes in an ice bath and centrifuged at 1000 g for 30 minutes at 4°C. The supernatant was used as an enzyme source. Enzyme activity was measured in a 10 mm path length cuvette using an incubation mixture consisting of 0.1 mL of enzyme source, 2.9 mL of 0.1 M buffer pH 8, 0.1 mL of chromogenic agent DTNB (5,5′-dithio-bis-2-nitrobenzoic acid), and 0.02 mL of freshly prepared ATChI (aetylthiocholine iodide) solution in distilled water. The change in optical density at 412 nm was recorded for 3 minutes after every 30 s interval at 25°C. Enzyme activity has been expressed as *μ*mole “SH” hydrolyzed min^−1^ mg^−1^ protein. For the estimation of kinetic constants of AChE, *in vitro* inhibition of the enzyme was carried out at different concentrations (3.0 × 10^−4^ M, 5.0 × 10^−4^ M, 7.0 × 10^−4^ M, and 1.0 × 10^−3^ M) of the substrate acetylthiocholine iodide. 

#### 2.3.2. Acid Phosphatase

Acid phosphatase (ACP) activity in the nervous tissue of *Lymnaea acuminata* was measured by the method of Bergmeyer [9] as modified by Singh and Agarwal [10]. Tissue homogenate (2%, w/v) was prepared in ice cold 0.9% NaCl and centrifuged at 5000 g for 15 minutes at 4°C. The supernatant was used as an enzyme source. 0.2 mL of enzyme source was added to 1.0 mL of acid buffer substrate (0.41 g citric acid, 1.125 g sodium citrate, and 165 mg 4-nitrophenyl phosphate sodium salt to 100 mL of double distilled water) preincubated at 37°C for 10 minutes. The incubation mixture was mixed thoroughly and incubated for 30 minutes at 37°C. 4 mL of 0.1 N NaOH was then added to the incubation mixture. The yellow colour, developed due to the formation of 4-nitrophenol, was determined colorimetrically at 420 nm. Standard curves were drawn with different concentrations of 4-nitrophenol. The ACP activity has been expressed as *μ*mole substrate hydrolyzed 30 min^−1^ mg^−1^ protein. For the determination of kinetic constants of acid phosphatase, *in vitro* inhibition of the enzyme was carried out at different concentrations (1.25 × 10^−5^ M, 1.8 × 10^−5^ M, 3.0 × 10^−5^ M, and 5.4 × 10^−5^ M) of the substrate 4-nitrophenyl phosphate.

#### 2.3.3. Alkaline Phosphatase

Alkaline phosphatase (ALP) activity in the nervous tissue of *Lymnaea acuminata* was measured by the method of Bergmeyer [9] as modified by Singh and Agarwal [10]. Tissue homogenate (2%, w/v) was prepared in ice cold 0.9% NaCl and centrifuged at 5000 g for 15 minutes at 4°C. The supernatant was used as an enzyme source. 0.1 mL of enzyme source was added to 1.0 mL of alkaline buffer substrate (375 mg glycine, 10 mg MgCl_2_.6H_2_O, 165 mg 4-nitrophenyl phosphate disodium salt in 42 mL of 0.1 N NaOH and a mixture was made up to 100 mL with double distilled water). The incubation mixture was mixed thoroughly and incubated for 30 minutes at 37°C. 10 mL of 0.02 N NaOH was then added to the incubation mixture. The yellow colour, developed due to the formation of 4-nitrophenol, was determined colorimetrically at 420 nm. Standard curves were drawn with different concentrations of 4-nitrophenol. The ALP activity has been expressed as *μ*mole substrate hydrolyzed 30 min^−1^ mg^−1^ protein. For the determination of kinetic constants of alkaline phosphatase, *in vitro* inhibition of the enzyme was carried out at different concentrations (1.2 × 10^−5^ M, 1.8 × 10^−5^ M, 3.0 × 10^−5^ M, and 5.4 × 10^−5^ M) of the substrate 4-nitrophenyl phosphate. 

#### 2.3.4. Protein

Protein estimation was carried out by the method of Lowry et al. [11] using bovine serum as a standard.

### 2.4. Statistical Analysis

Each experiment was replicated at least six times and results were expressed as mean ±SE of six replicates. Student's* t*-test was applied between control and treated groups to locate significant (*P* < .05) variations [12]. 

## 3. Results

### 3.1. In Vivo Inhibition of Enzymes

#### 3.1.1. Acetylcholinesterase

Table 1 shows that AChE activity in the nervous tissue of *Lymnaea acuminata* of control group was 0.73 *μ*mole “SH” hydrolyzed min^−1^ mg^−1^ protein. *In vivo* treatment of 40 and 80% of 24 and 96 h LC_50_ of trimyristin and myristicin caused significant (*P* < .05) inhibition in AChE activity in the nervous tissue of *Lymnaea acuminata*. Maximum inhibition of AChE activity (49% of control) was observed in snails exposed to 80% of 96 h LC_50_ of myristicin (Table 1).

#### 3.1.2. Acid Phosphatase

The acid phosphatase activity in the nervous tissue of *Lymnaea acuminata* of control group was 32.3 *μ*mole substrate hydrolyzed 30 min^−1^ mg^−1^ protein (Table 1). *In vivo* treatment of 40 and 80% of 24 and 96 hours LC_50_ of trimyristin and myristicin caused significant (*P *< .05) inhibition in ACP activity in the nervous tissue of *Lymnaea acuminata*. Maximum inhibition of ACP activity (44% of control) was observed in snails exposed to 80% of 96 hours LC_50_ of myristicin (Table 1).

#### 3.1.3. Alkaline Phosphatase

The alkaline phosphatase activity in the nervous tissue of *Lymnaea acuminata* of control group was 30.0 *μ*mole substrate hydrolyzed 30 min^−1^ mg^−1^ protein (Table 1). *In vivo* treatment of 40 and 80% of 24 and 96 h LC_50_ of trimyristin and myristicin caused significant (*P* < .05) inhibition in ALP activity in the nervous tissue of *Lymnaea acuminata*. Maximum inhibition of ALP activity (52% of control) was observed in snails exposed to 80% of 96 h LC_50_ of myristicin (Table 1).

### 3.2. In Vitro Inhibition of Enzymes

*In vitro* preincubation of 0.04, 0.07, 0.09, 0.1, and 0.15 mM of trimyristin and 0.01, 0.02, 0.03, 0.05, and 0.09 mM of myristicin caused significant (*P* < .05) dose dependent inhibition in AChE, ACP, and ALP activities (Table 2). IC_50_ values of trimyristin/myristicin against AChE, ACP, and ALP were 0.11, 0.16 and 0.18 / 0.03, 0.07 and 0.6 mM, respectively (Table 2). 

**Figure 1:**
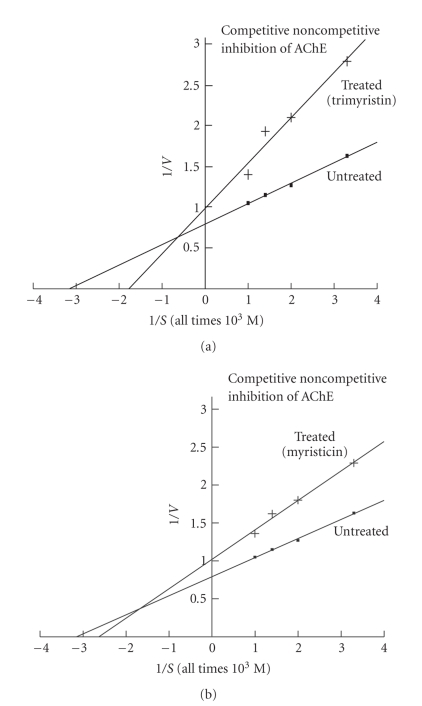
Lineweaver-Burk plots showing the effects of active molluscicidal components trimyristin (0.09 mM) (a) and myristicin (0.03 mM) (b) on the inhibition kinetics of acetylcholinesterase (AChE) activity in the nervous tissue of snail *Lymnaea acuminate*.

**Figure 2:**
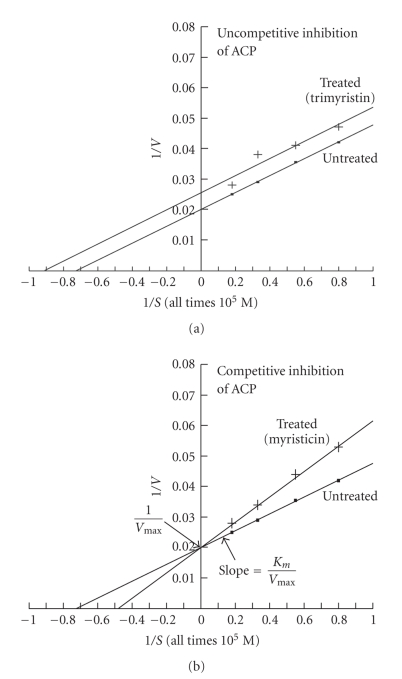
Lineweaver-Burk plots showing the effects of active molluscicidal components trimyristin (0.09 mM) (a) and myristicin (0.03 mM) (b) on the inhibition kinetics of acetylcholinesterase (ACP) activity in the nervous tissue of snail *Lymnaea acuminate*.

**Figure 3:**
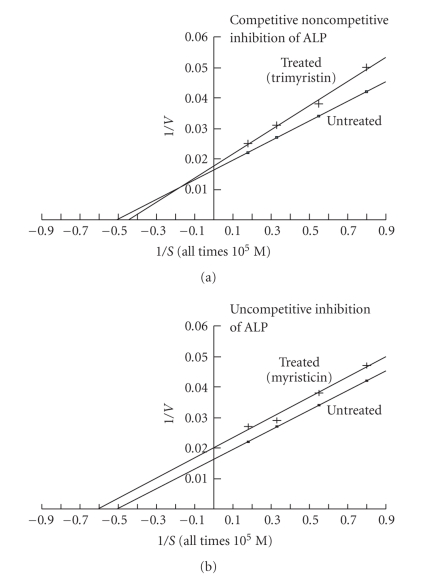
Lineweaver-Burk plots showing the effects of active molluscicidal components trimyristin (0.09 mM) (a) and myristicin (0.03 mM) (b) on the inhibition kinetics of acetylcholinesterase (ALP) activity in the nervous tissue of snail *Lymnaea acuminate*.

The  *K*_*m*_ and *V*_max_ values of uninhibited AChE were **3.17** × 10^−4^M and **1.25** *μ*mole “SH” hydrolyzed min^−1^ mg^−1^ protein, respectively (Table 3). *K*_*m*_, of trimyristin (Figure 1(a)) and myristicin (Figure 1(b)) inhibited AChE were **5.55** × 10^−4^M and **3.84** × 10^−4^ M, respectively. *V*_max _  values of trimyristin and myristicin inhibited AChE were **1.0** and **0.95** *μ*mole “SH” hydrolyzed min^−1^ mg^−1^ protein, respectively. *K*_*m*_ and *V*_max_ values of uninhibited ACP were **1.37** × 10^−5^ M and **50.00** *μ*mole substrate hydrolyzed 30 min^−1^ mg^−1^ protein, respectively (Table 3). *K*_*m*_ values of trimyristin (Figure 2(a)) and myristicin (Figure 2(b)) inhibited ACP were **1.09** × 10^−5^ M and **2.12** × 10^−5^ M, respectively. *V*_max_ values of trimyristin and myristicin inhibited ACP were **38.46** and **50** *μ*mole substrate hydrolyzed 30 min^−1^ mg^−1^ protein, respectively. *K*_*m*_ and *V*_max_ values of uninhibited ALP were **2.0** × 10^−5^ M and **62.50** *μ*mole substrate hydrolyzed 30 min^−1^ mg^−1^ protein, respectively (Table 3). *K*_*m*_ values of trimyristin (Figure 3(a)) and myristicin (Figure 3(b)) inhibited ALP were **2.27** × 10^−5^ M and **1.66** × 10^−5^ M, respectively. *V*_max_ values of trimyristin and myristicin inhibited ALP were **55.5** and **50** *μ*mole substrate hydrolyzed 30 min^−1^ mg^−1^ protein, respectively (Table 3).

## 4. Discussion

*In vivo* and *in vitro* sublethal treatments of trimyristin and myristicin caused a significant inhibition of AChE, ACP and ALP activities in the nervous tissue of *Lymnaea acuminata*. In *in vitro* condition, the activity of the enzyme is in the presence of drug. Extent of enzyme inhibition in *in vivo* and *in vitro* conditions is the almost same. In *in vivo* condition titer of the drug at action site may be low, yet it inhibited the enzyme up to the same extent as in *in vitro* condition. IC_50_ values of *trimyricitin* and myristicin clearly indicate that both are more potent inhibitors of AChE than ACP and ALP. AChE inhibition results in accumulation of acetylcholine at the nerve synapses, so that the postsynaptic membrane is in a state of permanent stimulation producing paralysis, ataxia, general lack of coordination in neuromuscular system, and eventual death [13]. It has been reported that n-hexane extract of *M. fragrans* seeds significantly inhibited AChE activity in brain of Swiss albino mice [14] and in *in vitro*, hydroalcoholic extracts of *M. fragrans* inhibited 50% of AChE activity at concentration of 100–150 *μ*g/mL using AChE obtained from bovine erythrocytes [15].

Acid phosphatase, a lysosomal enzyme [16], plays an important role in catabolism, pathological necrosis, autolysis, and phagocytosis [17]. Alkaline phosphatase plays a critical role in protein synthesis [18], shell formation, [19] other secretary activities [20], and transport of metabolites [21] in gastropods.

The kinetic study clearly indicates that the inhibition of AChE by trimyristin and myristicin is competitive noncompetitive, as *K*_*m*_ and *V*_max_ values of uninhibited and inhibited enzymes were different and slopes of inhibited and uninhibited AChE were also changed; both were not parallel to each other. Inhibitions of ACP by trimyristin and ALP, by myristicin are uncompetitive. It is evident from the Lineweaver-Burk plots that the slopes of trimyristin inhibited ACP, myristicin inhibited ALP and uninhibited ACP/ALP were not changed; both were parallel to each other, whereas the intercepts of inhibited and uninhibited ACP/ALP were changed. The *K*_*m*_ and *V*_max_, of uninhibited and inhibited enzymes were different. Inhibition of ACP by myristicin is competitive, as the *K*_*m*_ values of the uninhibited and inhibited myristicin enzymes were different and *V*_max_ of both were same, as evident from same intercept (1/*V*_max_) on the Y axis of Lineweaver-Burk plots. Inhibition of ALP by trimyristin is also competitive noncompetitive. 

Inhibition of AChE, ACP, and ALP by trimyristin and myristicin indicate, different types of inhibition kinetics. It seems that the molluscicidal components of *Myristica fragrans* kill the snails by inhibiting these enzymes in different ways.

## Figures and Tables

**Table 1:** *In vivo* effect of 24 and 96 h exposure to sublethal concentration of trimyristin and myristicin on acetylcholinesterase (AChE), acid phosphatase (ACP), and alkaline phosphatase (ALP) activities in the nervous tissue of *Lymnaea acuminata*.

Enzyme	Treatment	Enzyme activity				
		Control	40% of 24 h LC_50_	80% of 24 h LC_50_	40% of 96 h LC_50_	80% of 96 h LC_50_
AChE	Trimyristin	0.73 ± 0.0 (100)	0.66 ± 0.0* (90)	0.60 ± 0.0* (82)	0.53 ± 0.0*(73)	0.46 ± 0.0* (63)
	Myristicin	0.73 ± 0.0 (100)	0.57 ± 0.0* (78)	0.52 ± 0.0* (71)	0.45± 0.0* (62)	0.36 ± 0.0* (49)
ACP	Trimyristin	32.3 ± 0.1 (100)	28.0 ± 0.1*(87)	25.7 ± 0.3* (80)	23.7 ± 0.0* (73)	20.7 ± 0.2* (64)
	Myristicin	32.3 ± 0.1 (100)	26.0 ± 0.3* (80)	21.5 ± 0.0* (67)	17.9 ± 0.1* (55)	14.2 ± 0.1* (44)
ALP	Trimyristin	30.0 ± 0.1 (100)	27.5 ± 0.1* (92)	25.0 ± 0.1* (84)	21.4 ± 0.1* (71)	18.2 ± 0.2* (61)
	Myristicin	30.0 ± 0.1 (100)	22.8 ± 0.1* (76)	19.8 ± 0.1* (66)	17.7 ± 0.1* (59)	15.7 ± 0.2* (52)

Values are mean ±SE of six replicates. Values in parentheses indicate per cent of enzyme activity with control taken as 100%.

Concentrations (w/v) have been expressed as final concentration in aquarium water. Acetylcholinesterase activity, *μ*mole “SH” hydrolyzed min^−1^ mg^−1^ protein.

Acid phosphatase activity, *μ*mole substrate hydrolyzed 30 min^−1^ mg^−1^ protein.

Alkaline phosphatase activity, *μ*mole substrate hydrolyzed 30 min^−1^ mg^−1^ protein.

*Significant (*P* < .05) when *t*-test was used for locating difference between treated and control group of animals.

**Table 2:** *In vitro* effect of different concentrations (mM) of trimyristin and myristicin on the acetylcholinesterase (AChE), acid phosphatase (ACP), and alkaline phosphatase (ALP) activities in the nervous tissue of *Lymnaea acuminata*.

	Treatment				Enzyme activity			
		Control	0.04 mM	0.07 mM	0.09 mM	0.1 mM	0.15 mM	IC_50_ mM
AChE	Trimyristin	0.72 ± 0.0 (100)	0.60 ± 0.0* (83)	0.52 ± 0.0* (72)	0.45 ± 0.0* (63)	0.38 ± 0.0* (53)	0.18 ± 0.0* (25)	0.11
		Control	0.01 mM	0.02 mM	0.03 mM	0.05 mM	0.09 mM	
	Myristicin	0.72 ± 0.0 (100)	0.49 ± 0.0∗ (68)	0.42 ± 0.0* (58)	0.36 ± 0.0* (50)	0.29 ± 0.0* (40)	0.11 ± 0.0* (15)	0.03
		Control	0.04 mM	0.07 mM	0.09 mM	0.1 mM	0.15 mM	
ACP	Trimyristin	39.7 ± 0.1 (100)	35.5 ± 0.1* (90)	32.2 ± 0.0* (81)	27.9 ± 0.0* (70)	25.2 ± 0.1* (64)	8.1 ± 0.1* (20)	0.16
		Control	0.01 mM	0.02 mM	0.03 mM	0.05 mM	0.09 mM	
	Myristicin	39.7 ± 0.1 (100)	33.6 ± 0.2* (85)	29.0 ± 0.0* (73)	25.8 ± 0.0* (65)	22.7 ± 0.0* (57)	7.2 ± 0.2* (18)	0.07
		Control	0.04 mM	0.07 mM	0.09 mM	0.1 mM	0.15 mM	
ALP	Trimyristin	35.0 ± 0.2 (100)	33.0 ± 0.0* (94)	29.7 ± 0.2* (85)	26.7 ± 0.3* (76)	23.2 ± 0.4* (66)	5.6 ± 0.2* (16)	0.18
		Control	0.01 mM	0.02 mM	0.03 mM	0.05 mM	0.09 mM	
	Myristicin	35.0 ± 0.2 (100)	28.3 ± 0.0* (81)	25.1 ± 0.2* (72)	21.2 ± 0.3* (61)	18.9 ± 0.0* (54)	6.2 ± 0.1* (17.7)	0.06

Values are mean ±SE of six replicates. Values in parentheses indicate per cent of enzyme activity with control taken as 100%.

Concentrations (mM) have been expressed as final concentration in the incubation mixture present in the cuvette.

Acetylcholinesterase activity, *μ*mole “SH” hydrolyzed min^−1^ mg^−1^ protein.

Acid phosphatase activity, *μ*mole substrate hydrolyzed 30 min^−1^ mg^−1^ protein.

Alkaline phosphatase activity, *μ*mole substrate hydrolyzed 30 min^−1^ mg^−1^ protein.

*Significant (*P* < .05) when *t*-test was applied between treated and control groups.

**Table:** (a) Michaelis-Menten constant *K*_*m*_ and *V*_*max*_ of different enzymes were calculated from Lineweaver-Burk Plots (1/V versus 1/S).

Enzymes	Untreated (Control)		Treated (Trimyristin)		Treated (Myristicin)	
	*K*_*m*_	*V*_max _	*K*_*m*_	*V*_max _	*K*_*m*_	*V*_max _
AChE	3.1 × 10^−4^	1.2	5.5 × 10^−4^	1.0	3.8 × 10^−4^	0.95
ACP	1.3 × 10^−5^	50.0	1.0 × 10^−5^	38.4	2.1 × 10^−5^	50.00
ALP	2.0 × 10^−5^	62.5	2.2 × 10^−5^	55.5	1.6 × 10^−5^	50.00

**Table:** (b) Michaelis-Menten constant *K*_*m*_ and *V*_*max*_ of different enzymes were calculated from nonlinear regression (V versus S).

Enzymes	Untreated (Control)		Treated (Trimyristin)		Treated (Myristicin)	
	*K*_*m*_	*V*_max _	*K*_*m*_	*V*_max _	*K*_*m*_	*V*_max _
AChE	2.9 × 10^−4^	1.1	4.9 × 10^−4^	0.84	3.2 × 10^−4^	0.80
ACP	0.9 × 10^−5^	40	0.8 × 10^−5^	33	1.9 × 10^−5^	46
ALP	1.8 × 10^−5^	56	1.3 × 10^−5^	45	1.3 × 10^−5^	47
